# Aneurysmal subarachnoid hemorrhage with PFBC and beta thalassemia: a case report

**DOI:** 10.1186/s12883-023-03072-2

**Published:** 2023-01-23

**Authors:** Kuangyang Yu, Jinwei Pang, Xiaobo Yang, Jianhua Peng, Yong Jiang

**Affiliations:** 1 Department of Neurosurgery, the Affiliated Hospital of Southwest Medical University, Luzhou, 646000 China; 2 Laboratory of Neurological Diseases and Brain Function, the Affiliated Hospital of Southwest Medical University, Luzhou, 646000 China; 3 Sichuan Clinical Research Center for Neurosurgery, the Affiliated Hospital of Southwest Medical University, Luzhou, 646000 China; 4 Institute of Epigenetics and Brain Science, Southwest Medical University, Luzhou, 646000 China; 5 Academician (Expert) Workstation of Sichuan Province, the Affiliated Hospital of Southwest Medical University, Luzhou, 646000 China

**Keywords:** Intracranial aneurysm, Primary familial brain calcification, Fahr’s disease, Intracranial haemorrhage, Beta-thalassemia

## Abstract

**Background:**

Primary familial brain calcification (PFBC), habitually called Fahr’s disease, is characterized by bilateral calcification of the basal ganglia, accompanied by extensive calcification of the cerebellar dentate nucleus, brainstem cerebrum, and cerebellum at the grey-white matter junction. However, there are few reports about PFBC with aneurysmal subarachnoid hemorrhage (aSAH) and thalassemia.

**Case presentation:**

We describe a patient admitted to the hospital with an acute deterioration in the level of consciousness with no history of neuropsychiatric features or movement disorders. After computed tomography (CT) and CT angiography (CTA), the patient was diagnosed with PFBC, accompanied by aneurysmal subarachnoid haemorrhage (aSAH), intracranial haemorrhage (ICH), and hemoglobin electrophoresis suggested beta-thalassemia. This patient underwent craniotomy aneurysm clipping and intracranial hematoma removal.

**Conclusions:**

For patients with PFBC, we should pay attention to their blood pressure and intracranial vascular conditions. The CTA is necessary to clarify the cerebrovascular conditions of the patient, especially when combined with hypertension and persistent headache or other related prodromal symptoms of cerebrovascular disease.

## Background

Primary familial brain calcification (PFBC) [1], habitually called Fahr’s disease, was described by Karl Theodor Fahr in 1930 [2]. Computed tomography (CT) imaging shows extensive bilateral basal ganglia calcifications in the brain. The clinical symptoms of PFBC may include movement disorders or neuropsychiatric manifestations [3], such as parkinsonism, cognitive impairment, psychosis, seizures and chronic headaches [4–6]. Patients are usually diagnosed with PFBC in the hospital due to the appearance of these symptoms. Although the disease is a calcification of the brain parenchyma, there are few reports of its association with cerebrovascular disease.

In this paper, we present a case with PFBC complicated with aneurysmal subarachnoid haemorrhage (aSAH) and multiple intracerebral haemorrhages (ICH), as well as β thalassemia. Based on the previous literature, we also discussed the location and possible cause of intracranial haemorrhage in PFBC patients.

## Case presentation

A 55-year-old woman was admitted to the Neurointensive Care Unit because of loss of consciousness for nearly 10 h. She was previously healthy and developmentally normal. The patient had a history of hypertension for more than four years and regularly took medication to control her blood pressure. Her son was previously diagnosed with beta-thalassemia, but she has not undergone any head CT or other examination before. In addition, this patient had no significant medical history, such as cardiovascular and cerebrovascular diseases, or any neurological symptoms. This patient also did not have the typical clinical manifestations (neuropsychiatric features and movement disorders) of PFBC in the past, and there were no similar cases in her family.

**Fig. 1:**
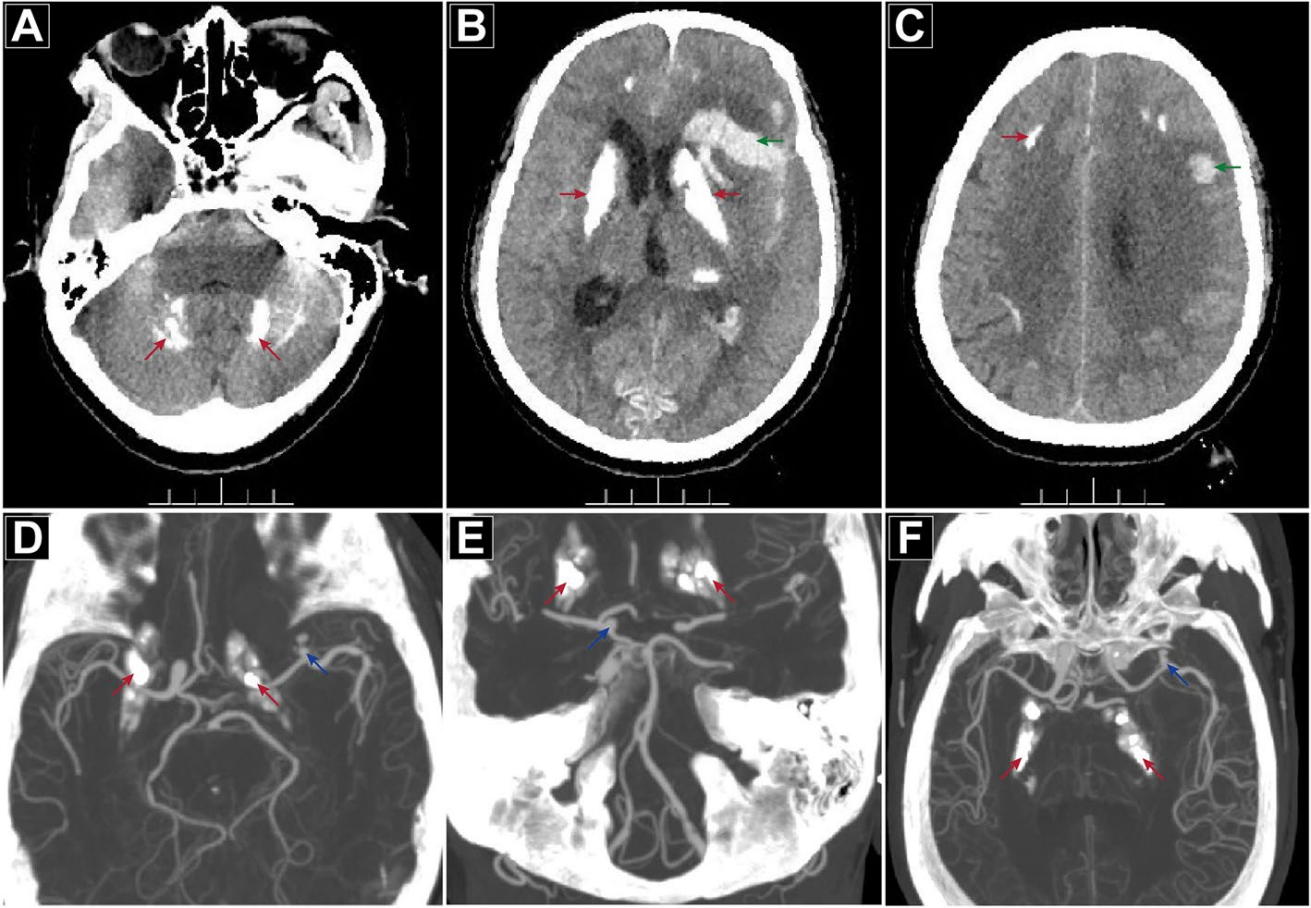
CT scan and CTA at admission

Neurological examination on admission showed Glasgow Coma Scale (GCS): 5; Hunt-Hess Scale: III; Modified Fisher Scale: IV; National Institute of Health Stroke Scale (NIHSS): 31. The pupils were both 2.5 mm, equal and round, with normal adjustment reflection but a slow light response. In addition to the stiff neck, no obvious positive signs were found on the other physical examinations. A CT scan confirmed the multiple intracranial symmetrical calcifications, SAH, and left frontotemporal-lateral and paraventricular haemorrhage (Fig. 1A-C). CT angiography (CTA) showed an aneurysm of the left middle cerebral artery M1 segment terminal (Fig. 1D-F). Hemoglobin electrophoresis suggested beta-thalassemia. Repeated examinations of parathyroid hormone, thyroid hormone and related antibodies showed no abnormality, autoantibody profiles and immunoglobulins were normal, IgG and IgA levels were normal, and there were no disorders of calcium and phosphorus or trace elements metabolism. There was a slight increase in her serum IgM level, and her 25-hydroxyvitamin D level was low.

Combined with the patient’s medical history and imaging features, a diagnosis of PFBC, aSAH, ICH and beta-thalassemia was considered. During hospitalization, this patient underwent craniotomy aneurysm clipping and intracranial hematoma removal. Intraoperatively, the aneurysm size was about 3 × 5 mm. 16 days after admission, her postoperative condition improved significantly, and she was discharged from the hospital for further rehabilitation. The patient's Modified Rankin Scale (mRS) score at discharge was IV, and the 3 months follow-up mRS was II. There were no adverse or unanticipated events.

## Discussion and conclusion

PFBC is a rare neurodegenerative disorder characterized by symmetrical and bilateral calcifications in the basal ganglia. The calcifications may also occur in the dentate nucleus, thalamus, and cerebral cortex. Both nonfamilial and familial cases of PFBC have been reported, predominantly inherited in an autosomal-dominant fashion [5]. It may also be related to calcium and phosphorus metabolism and endocrine changes [7].

Previous studies has shown that changes of some genes, such as solute carrier family 20 member 2 (*SLC20A2*), platelet-derived growth factor receptor alpha (*PDGFRB*), platelet-derived growth factor subunit B (*PDGFB*), xenotropic and polytropic retrovirus receptor 1 (*XPR1*) and myogenesis regulating glycosidase (*MYORG*), are associated with the pathogenesis of PFBC [8]. The pathogenic changes of *SLC20A2*, *XPR1*, *PDGFB* and *PDGFRB* are the reasons for the autosomal dominant inheritance mode of the disease [9–12]. Furthermore, the biallelic mutation of the *MYORG* gene determines the cause of autosomal recessive PFBC [13, 14]. However, these genes can only explain less than half of all PFBC cases [8]. The patient's parents have no clinical manifestations or related diseases during the collection of medical history, which precludes the possibility of dominant inheritance. Next-generation sequencing or genotype testing was not performed in this patient, but according to previous studies, the CT images (no pontine calcification) and pre-admission conditions (no dysarthria, movement signs, etc.) do not support the patient as *MYORG-*mediated PFBC [15].

PFBC has a variety of clinical manifestations, predominantly with neuropsychiatric features and movement disorders [5]. Neuropsychiatric features include cognitive impairment, depression, hallucinations, delusions, manic symptoms, anxiety, schizophrenia-like psychosis, and personality changes [16, 17]. Other clinical features include parkinsonism, ataxia, headache, seizures, vertigo, stroke-like events, orthostatic hypotension, tremor, dysarthria, and paresis [18]. Interestingly, PFBC often begins with these clinical features, leading the patients to go to the hospital and therefore be diagnosed with PFBC. However, this patient did not have neuropsychiatric features and movement disorders as her primary manifestations.

Koutsis et al. reported a case of β-thalassemia major combined with PFBC [19]. In that case, long-term transfusions created iron overload, which may cause hypoparathyroidism in thalassemia patients [20]. Besides, previous studies have confirmed that hypoparathyroidism, infection and systemic lupus erythematosus can lead to the formation of calcifications. In our case, she had no history of anemia-related symptoms or blood transfusion, nor did she have any associated endocrine changes. She also did not have calcium, phosphorus, or trace elements metabolism changes. Although 25-hydroxyvitamin D changes and calcifications have been reported to cause intracranial calcification, vitamin supplementation is sufficient for menopausal women who have low levels of 25-hydroxyvitamin D [21]. Therefore, there might be other reasons for the formation of intracranial calcifications in this patient. Notably, this patient did not have endocrine changes, which may have led to her calcifications being smaller than in previous cases, and their small size might be why the patient had no clinical manifestations.

Previously reported literature may shed light on the possible consequences of vascular calcification in these patients. A 65-year-old female patient with PFBC was reported to have a rare site cerebral haemorrhage because of the massive calcification of cerebral blood vessels in the basal ganglia (the most common site of cerebral haemorrhages) that may have prevented the occurrence of hypertensive cerebral hemorrhage [22]. In a report of a 36-year-old male patient with PFBC and ischemic stroke, it was proposed that the underlying pathogenic process of PFBC may lead to extensive calcium and mineral deposits in diseased vessels [23]. Uygur et al. implemented intracranial blood flow testing in the patient and recorded decreased blood flow in calcified areas, which may have led to further focal ischemic changes [24]. Neuropathology has proved that calcifications are observed in the media of medium and small calibre arteries, arterioles, and capillaries in PFBC, leading to luminal obstruction [25]. Further research confirms that alcium-phosphate accumulation initiates in neurovascular unit (including neurons, astrocytes, microglia, pericytes and endothelial cells) in PFBC patients and mouse models [8]. Calcium deposits can be a double-edged sword since a certain degree of calcification may be a favorable manifestation of blood vessels, preventing bleeding. However, severe calcification can lead to obstruction of the blood supply.

Based on these findings, we speculate that PFBC may be due to the formation of abnormal intracranial vascular calcification, which leads to a decrease in blood flow in the calcified area. This then leads to ischemic manifestations in the calcified area and an increased likelihood of bleeding elsewhere. Further clinical cohort studies are necessary to confirm our speculation. Regarding β-thalassemia, the occurrence of β-thalassemia and PFBC is unclear, and there are very few reports. We need more cases to confirm the relationship between β-thalassemia and PFBC and the possible underlying mechanism.

Patients with PFBC may be complicated by hypertension and intracranial aneurysm and other related diseases. Existing examples and evidence have confirmed that abnormal vascular calcification can have severe effects on blood vessels. Although the related factors and causes are unknown, we should pay attention to the blood pressure and intracranial blood vessels of PFBC patients. If there are enough cases, further mechanisms research on cerebrovascular disease, cerebral blood flow, and PFBC-related factors should be conducted.

In conclusion, our case provides clear images of the relative positions of PFBC and a ruptured intracranial aneurysm. This is the first reported case of a patient with PFBC associated with aSAH, ICH, and β-thalassemia. For patients with PFBC, we should pay attention to their blood pressure and intracranial vascular conditions, especially when combined with hypertension and persistent headache or other related prodromal symptoms of cerebrovascular disease. The association between PFGC pathogenic genotype and thalassemia is a point that we should further explore.

## Data Availability

Data presented are available on request from the corresponding author.

## Abbreviations

aSAH: Aneurysmal subarachnoid hemorrhage
CT: Computed tomography
CTA: CT angiography
ICH: Intracranial hemorrhage
GCS: Glasgow Coma Scale
mRS: Modified Rankin Scale
MYORG: Myogenesis regulating glycosidase
NIHSS: National Institute of Health Stroke Scale
PFBC: Primary Brain calcification
PDGFB: Platelet-derived growth factor subunit B
PDGFRB: Platelet-derived growth factor receptor alpha
SLC20A2: Solute carrier family 20 member 2
XPR1: Xenotropic and polytropic retrovirus receptor 1
